# Supplementary material for: A comprehensive economic optimization methodology of divided wall columns for biopolyol separation
Source: R Soc Open Sci. 2020 Apr 15;7(4):191748. doi: 10.1098/rsos.191748 (PMC7211852; doi:10.1098/rsos.191748)
Supplement: Supplementary information [file rsos191748supp1.docx]

**Supplementary information**

**A comprehensive economic optimization methodology of divided wall columns for biopolyol separation.**

**Tao Chen, Lingjuan Lv, Yuanzhi Chen, Peng Bai**

*Department of Pharmaceutical Engineering, School of Chemical Engineering and Technology,* *Tianjin University, Tianjin 300350, P. R. China*

*Key Laboratory of Systems Bioengineering (Ministry of Education), Tianjin University, Tianjin, 300072,* *P. R. China*

*Corresponding author. E-mail: [chentao123@tju.edu.cn](mailto:chentao123@tju.edu.cn)

**Table S1** Binary interactive parameters of NRTL model

| Component i | Component j | A_ij_ | A_ji_ | B_ij_/K | B_ji_/K | C_ij_ |
| --- | --- | --- | --- | --- | --- | --- |
| 1,2-PG | EG | -1.28 | -0.43 | 995.38 | -136.44 | 0.30 |
| 1,2-PG | 1,3-PG | -1.54 | -0.65 | 465.27 | 636.46 | 0.50 |
| EG | 1,3-PG | -2.95 | 1.74 | 1002.23 | -246.97 | 0.27 |


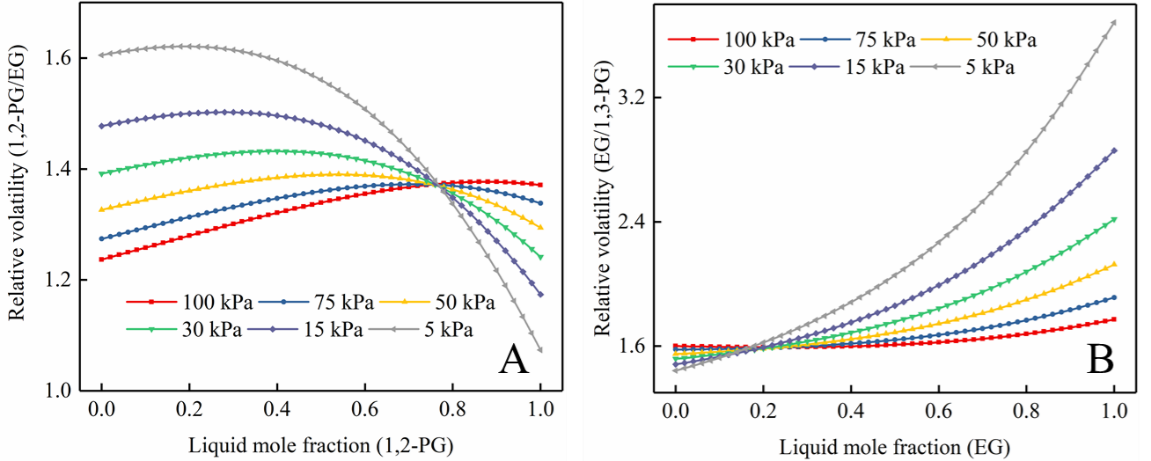


**Fig S1.** Relative volatility of (A) 1,2-PG/EG, (B) EG/1,3-PG at different pressures.


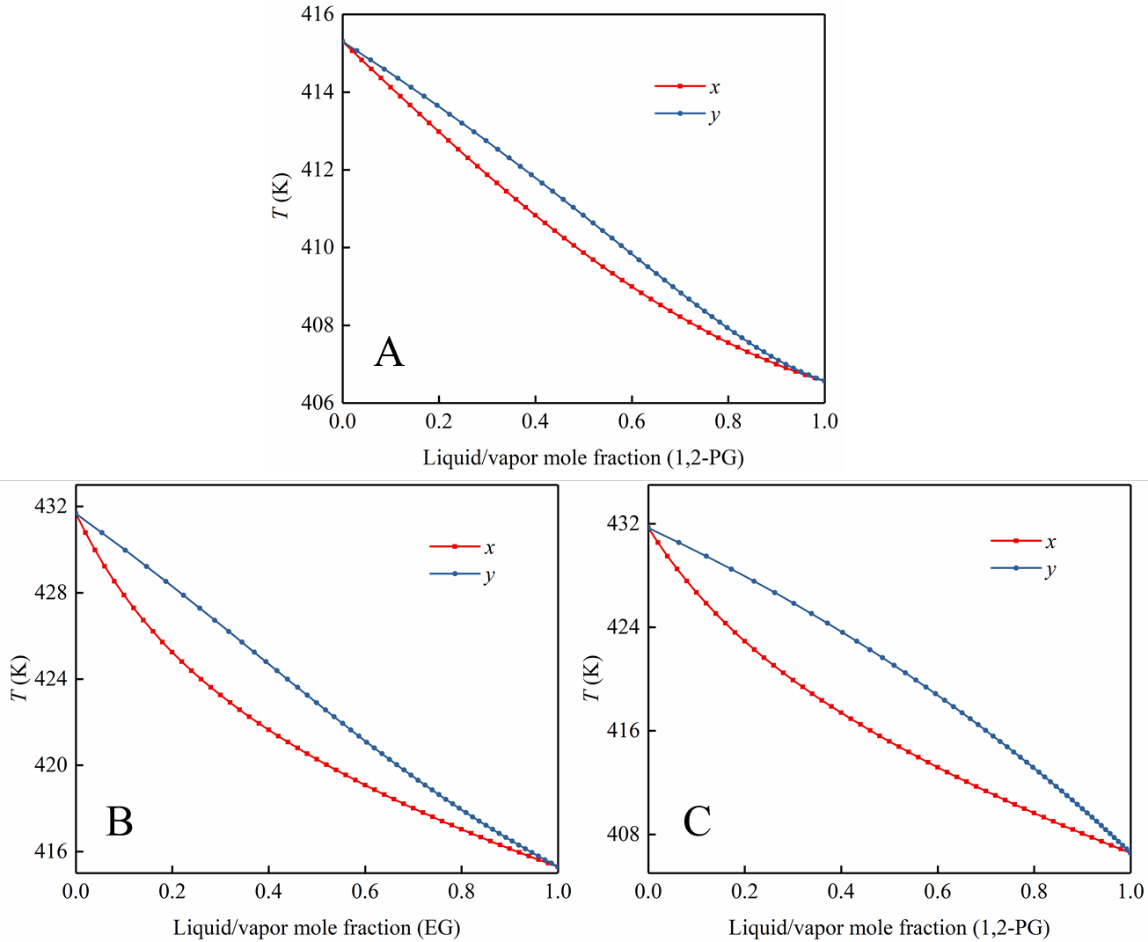


**Fig S2.** VLE data for (A) 1,2-PG + EG, (B) EG + 1,3-PG, (C) 1,2-PG + 1,3-PG at 15 kPa.

**Table S2** Second-order polynomial model of response fitting groups of variables

| Responses | Variables | Fitting models in term of code factors | Value P | R^2^ |
| --- | --- | --- | --- | --- |
| TAC saving (%) | A (*q*), B (*r_F_*), C (*r_S_*) | 39.55+0.11A+0.063B-0.13C-0.050AB-  0.025AC-0.18BC-0.48A^2^-0.43B^2^-0.20C^2^ | <0.0001 | 0.989 |
|  | A (N1), B (N2), C (N3), D (N4) | 39.29+0.11A+1.1B+1.51C-0.28 D-0.42AB-0.58AC-0.60AD-0.72 BC-0.32BD-0.080CD  -0.29A^2^-1.44 B^2^-1.19C^2^-0.44D^2^ | <0.0001 | 0.992 |
|  | A (*S_L_*), B (*S_G_*), C (*R*) | 39.660.85A+0.41B1.24C+1.57AB+1.28AC  +0.26BC+0.44A^2^-3.22B^2^-2.74C^2^ | <0.0001 | 0.996 |

**Table S3** Composition and Flowrate of streams in biopolyol divided wall column.

|  | | Streams | | |
| --- | --- | --- | --- | --- |
|  | | D | S | W |
| Composition | 1,2-PG | 0.991 | 0.0047 | 0 |
|  | EG | 0.009 | 0.992 | 0.003 |
|  | 1,3-PG | 0 | 0.0032 | 0.997 |
| Flowrate (kmol/h) | | 29.8 | 40.1 | 30.1 |
